# Genetic Dissection of the *Drosophila melanogaster* Female Head Transcriptome Reveals Widespread Allelic Heterogeneity

**DOI:** 10.1371/journal.pgen.1004322

**Published:** 2014-05-08

**Authors:** Elizabeth G. King, Brian J. Sanderson, Casey L. McNeil, Anthony D. Long, Stuart J. Macdonald

**Affiliations:** 1 Department of Ecology and Evolutionary Biology, University of California Irvine, Irvine, California, United States of America; 2 Department of Molecular Biosciences, University of Kansas, Lawrence, Kansas, United States of America; 3 Department of Biology, Newman University, Wichita, Kansas, United States of America; Georgia Institute of Technology, United States of America

## Abstract

Modern genetic mapping is plagued by the “missing heritability” problem, which refers to the discordance between the estimated heritabilities of quantitative traits and the variance accounted for by mapped causative variants. One major potential explanation for the missing heritability is allelic heterogeneity, in which there are multiple causative variants at each causative gene with only a fraction having been identified. The majority of genome-wide association studies (GWAS) implicitly assume that a single SNP can explain all the variance for a causative locus. However, if allelic heterogeneity is prevalent, a substantial amount of genetic variance will remain unexplained. In this paper, we take a haplotype-based mapping approach and quantify the number of alleles segregating at each locus using a large set of 7922 eQTL contributing to regulatory variation in the *Drosophila melanogaster* female head. Not only does this study provide a comprehensive eQTL map for a major community genetic resource, the *Drosophila* Synthetic Population Resource, but it also provides a direct test of the allelic heterogeneity hypothesis. We find that 95% of *cis*-eQTLs and 78% of *trans*-eQTLs are due to multiple alleles, demonstrating that allelic heterogeneity is widespread in *Drosophila* eQTL. Allelic heterogeneity likely contributes significantly to the missing heritability problem common in GWAS studies.

## Author Summary

For traits with complex genetic inheritance it has generally proven very difficult to identify the majority of the specific causative variants involved. A range of hypotheses have been put forward to explain this so-called “missing heritability”. One idea—allelic heterogeneity, where genes each harbor multiple different causative variants—has received little attention, because it is difficult to detect with most genetic mapping designs. Here we make use of a panel of *Drosophila melanogaster* lines derived from multiple founders, allowing us to directly test for the presence of multiple alleles at a large set of genetic loci influencing gene expression. We find that the vast majority of loci harbor more than two functional alleles, demonstrating extensive allelic heterogeneity at the level of gene expression and suggesting that such heterogeneity is an important factor determining the genetic basis of complex trait variation in general.

## Introduction

Uncovering the genetic basis of quantitative phenotypes is a central, yet unresolved problem in biology. There is a major discrepancy between the heritability estimates of most quantitative traits and the amount of heritable variation accounted for by all variants localized to a causative site. This phenomenon is often referred to as the “missing heritability” problem. Several hypotheses have been offered as possible explanations, including widespread epistasis [1], the infinitesimal model (many, very small effect loci influencing the phenotype of interest that are difficult to detect statistically) [2]–[4], rare alleles of large effect, that are also statistically difficult to detect [5]–[7], and widespread allelic heterogeneity (many independent effects segregating at each causative locus) [7]. This quest to understand the genetic basis of complex traits has given rise to a community-based strategy of creating freely-available genetic resource populations in model organisms such as mice [8]–[10], *Arabidopsis thaliana* [11], [12], maize [13]–[16], and *Drosophila melanogaster* [17]–[20]. Those organisms with the greatest genetic resources and with a community of researchers focused on a single system provide a logical starting point toward finding the missing heritability associated with quantitative phenotypes. In addition, the experimental designs of some of these resources are well suited to test different hypotheses for the sources of missing heritability. For example, Bloom et al. [21] used a large segregant pool from a two line yeast cross to demonstrate that epistasis is not a major contributor to the heritability of most traits. In particular, resources that have a well-defined multi-haplotype structure can be used to identify the extent of allelic heterogeneity [22] owing to the ability to estimate trait means for each haplotype at each mapped QTL. By focusing effort on these community resources, the hope is that we will gain a better understanding of the causes of missing heritability problem.

Much of the genetic variation underlying whole organism phenotypes is thought to be due to regulatory variation, i.e., variants influencing gene expression [23]–[26]. Causative loci are linked to whole organism phenotypes through the transcriptome, an interrelated network of transcripts whose abundances influence the resulting phenotype. The transcript abundances of most genes are quantitative traits themselves and have heritabilities comparable to typical whole-organism phenotypes [24], [26], [27]. Increasingly, expression quantitative trait locus (eQTL) mapping is being used to identify the source of genetic variation in transcript abundances with the ultimate goal of linking variation at the nucleotide level to variation in gene expression and to variation in visible phenotypes. Expression QTL studies have shown that most genes have local (*cis*) eQTL that tend to be located near the transcription start site and to be of fairly large effect. Distant regulatory effects (*trans*-eQTL) are more difficult to identify, likely because they are more numerous and are of smaller average effect, leaving a great deal of variation in transcript abundance unexplained [23], [24], [26], [27]. There is a growing movement toward identifying the causative quantitative trait nucleotides (QTN) underlying *cis*-eQTL, often with the assumption there is a single causative site [28]–[30]. However, if most eQTL harbor allelic heterogeneity [31], identifying a single causative variant will cause researchers to miss a significant portion of the genetic variation [7].

Here we describe transcriptome-wide mapping in female head tissue in the *Drosophila* Synthetic Population Resource (DSPR) [17], [18], one of the major genetic reference panels in the *Drosophila* model system. Our goals are two-fold. First, we aim to provide a comprehensive map of *cis*- and *trans*-eQTL for female head tissue in the DSPR. A key advantage of genetic reference panels is the potential to integrate phenotypes measured at multiple levels on genetically identical individuals. Incorporating eQTL data with visible phenotype data can increase mapping power and help users identify candidate genes [9], [23], [25], [32]. Second, we use the large set of discovered eQTL to quantify the number of alleles segregating at each causative locus, providing an evaluation of the degree of allelic heterogeneity at both *cis*- and *trans*-eQTL. The hypothesis that allelic heterogeneity is prevalent in quantitative traits has not been tested directly, in part because it is difficult to do so using a genome-wide association (GWAS) framework. Within loci, linkage disequilibrium makes it very difficult to distinguish between two SNPs tagging two independent causative sites versus a single causative site. In addition, the step-wise regression approaches used, for example [2], [33], to identify multiple SNPs in a gene region associated with a phenotype lack power. The result is that the majority of GWAS that have identified multiple SNPs at a single locus using conditional analysis rarely identify more than two such SNPs despite very large sample sizes e.g. [2] but see [33]. In contrast, mapping in the DSPR and other multi-parental advanced generation intercross mapping panels take a haplotype based approach, providing a natural way to distinguish between multiple alleles at each QTL and a way to ascertain the potential contribution of allelic heterogeneity to the missing heritability problem.

## Results and Discussion

We mapped genome-wide expression variation using *trans*-heterozygote F1 individuals from 596 crosses between DSPR population A (pA) females and population B (pB) males, thus avoiding mapping variation for inbreeding depression. Gene expression was assayed using Nimblegen 12×135 K arrays, and we analyzed the resulting data using a custom data analysis pipeline (see methods) to identify all significant eQTL.

### The female head eQTL map

**Figure 1:**
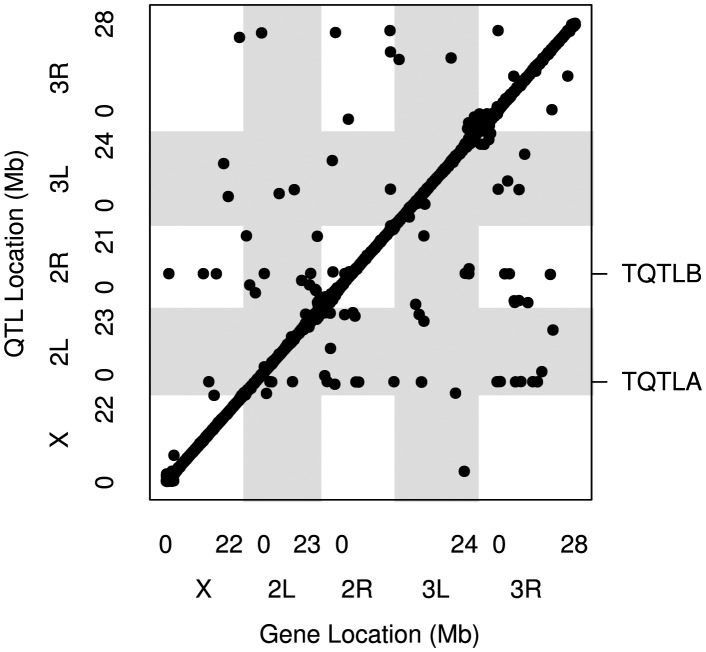
The locations of all mapped eQTL. The location of the transcripts whose expression measures are mapped are along the x axis and the location of the corresponding eQTL peaks are along the y axis. Points falling along the diagonal indicate eQTL mapping to the same location as transcripts (*cis*) while those off the diagonal map to different locations (*trans*). The clusters of points near each centromere are within 1.5 cM of the target gene (*cis*) but are further away in physical distance due to the low recombination rate and lower mapping resolution in this region. Grey and white shading denote the different chromosome arms. The two *trans* hotspots we identified are labeled on the right axis (See Figure 4).

We identified a total of 7922 eQTLs corresponding to 7850 transcripts out of a total of 11064 transcripts tested (Figure 1). Details for all eQTLs are in Table S1. Of these, 7704 transcripts were associated with a single *cis*-eQTL, 71 were associated with both *cis*- and *trans*-eQTL, and 75 were associated with only *trans*-eQTL. A small percentage of eQTLs (∼7%; Table 1) were associated with only a single recombinant inbred line (RIL) population (pA or pB; see methods), but for most eQTL fitting both pA and pB was necessary to explain the eQTL signal, indicating that causative variants were present in both populations.

**Table 1:** Numbers of *cis*- and *trans*-eQTL mapped for different models.

	*cis*	*trans*	Total
Model			
pA+pB	7220	121	7341
pA only	303	7	310
pB only	252	19	271
Total	7775	147	7922

**Figure 2:**
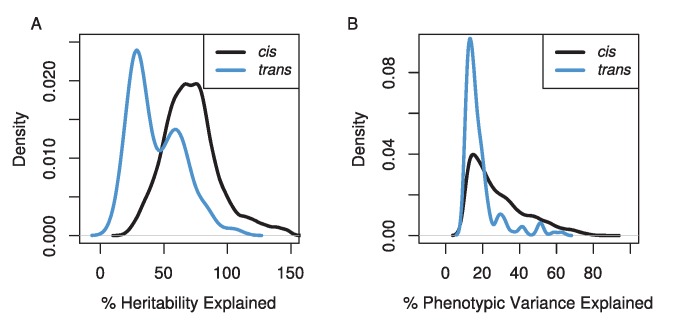
The distributions of the percentage of the genetic variation (A) and phenotypic variation (B) explained for *cis*- (black line) and *trans*- (blue line) eQTL. Estimates of the percentage of genetic variance explained can be greater than 100% due to underestimates of heritability for transcripts and/or overestimates of effect sizes of eQTL.

The amount of variation explained by our mapped eQTLs was high (Figure 2), though our stringent, experiment-wise permutation-based correction for multiple tests severely limits our ability to detect QTL of small effect. Not surprisingly, the variance explained by *cis*-eQTLs was higher than *trans*-eQTLs [24]. Our *cis*-eQTLs explained a median of 24% of the phenotypic variance, and 855 eQTL explained more than 50% of the phenotypic variance. Using our heritability estimates for each transcript abundance, we estimated the percentage of the heritability each eQTL explained. The median for the percent heritability explained by each eQTL was 73%. Our *trans*-eQTLs explained lower levels of variance, the median phenotypic variance explained was 15%, and the median percent heritability explained was 38%. However, if heritability values are underestimated, and/or we overestimate the effects of eQTLs (which is likely due to the Beavis effect [34]), these values will be inflated. This effect is obvious for the set of eQTL estimated to explain greater than 100% of the heritability (Figure 2A).

**Figure 3:**
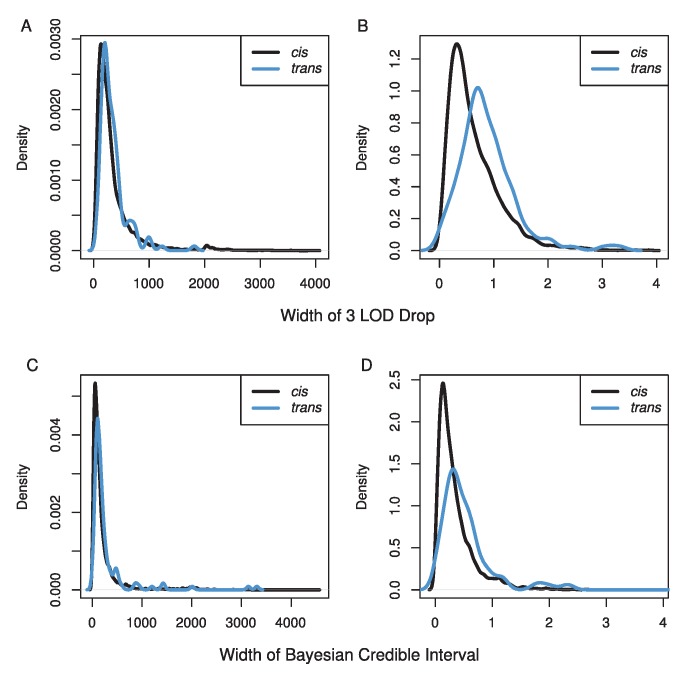
Distributions of the width of our confidence intervals for eQTL in A & C) physical distance (kb) and B & D) genetic distance (cM) using either a 3 LOD drop (A & B) or the Bayesian credible interval (C & D). Black lines show CIs for *cis*-eQTL while blue lines show CIs for *trans*-eQTL.

Our mapping resolution was high (Figure 3). We used two methods for estimating confidence intervals, a 3 LOD drop and the Bayesian credible interval. We excluded confidence intervals that spanned centromeres or occurred near telomeres, because these tend to cover very large physical distances (7% of eQTLs). The Bayesian credible intervals tended to be narrower than 3 LOD drops (median BCI = 110 kb, 0.25 cM; median 3 LOD drop = 240 kb, 0.51 cM), but the range was larger for BCIs (BCI: 0–4.5 Mb, 0–6.5 cM; 3 LOD drop: 20 kb–4.0 Mb, 0.001–3.9 cM). The median number of genes within *cis*-eQTL CIs was 32 (range 1–551), and within *trans*-eQTL CIs, the median was 44 (range: 5–479).

We have provided a comprehensive map of eQTLs for female head tissue in the *Drosophila* model system within the constraints of our statistical power. There is little doubt many smaller effect eQTLs exist that we were not able to identify given our conservative statistical threshold. Our use of *trans*-heterozygote individuals means that we not only avoid the effects of inbreeding depression, but we have also obtained estimates for all eQTL for both pA and pB DSPR populations. Overall, our results confirm what many other researchers have observed, widespread large effect *cis*-eQTLs and smaller effect *trans*-eQTLs [23], [24], [26], [27]. One of the major advantages of a stable genetic panel is the ability to measure multiple traits at multiple levels on genetically identical individuals, which allows for the potential to combine these sources of data to identify causative genes [9], [23], [25], [32]. We expect this dataset to be very useful to DSPR users, particularly those interrogating phenotypes measured in females with relevance to neuroanatomy or behavior. All of the raw and analyzed data are freely available at http://FlyRILs.org/Data. The data have also been deposited in NCBI's Gene Expression Omnibus [35] and are accessible through GEO Series accession number GSE52076.

### *Trans*-eQTL hotspots

**Figure 4:**
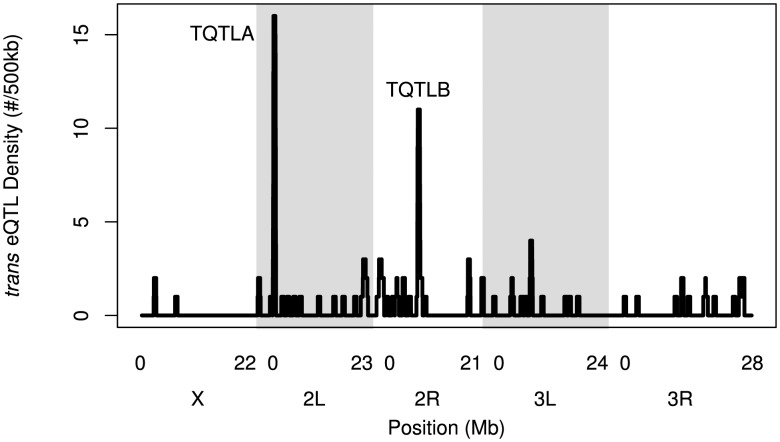
The density of *trans*-eQTLs along the genome. Density is the number of genes (not transcripts) with a *trans*-eQTL per 500 kilobases. Grey and white shading denote the different chromosome arms. Potential hotspots (regions with a density greater than 5) are noted.

We identified regions of the genome associated with a high *trans*-eQTL density to identify eQTL regulating the expression of several other genes (*trans* hotspots). There were two regions of high *trans*-eQTL density, TQTLA and TQTLB (Figure 4; Table 2). These clusters regulate several genes distributed throughout the genome, as is apparent in Figure 1. We used a gene ontology term finder [36] to determine whether the sets of genes regulated by these *trans*-eQTL were related to a common process. The set of 16 genes regulated by TQTLA showed enrichment for circadian rhythm of gene expression (2 of the 16 genes regulated by TQTLA; P = 0.0007). We used principal components analysis on the set of 16 genes to create a composite variable. All 16 genes load fairly evenly on the first principal component (absolute value range: 0.08–0.20). We then correlated this composite variable with expression measures for each gene in the TQTLA region to identify possible candidate genes. The gene *timeless (tim)* was highly correlated with the TQTLA composite variable (r = 0.90), and it does have a significant *cis*-eQTL. All other genes in the interval had a correlation with an absolute value of less than 0.5. Additionally, after correlating the expression of each of the 16 transcripts regulated by TQTLA with the expression of all genes in the TQTLA region, timeless showed the maximum pairwise correlation in all 16 cases (absolute value of correlation range:0.35–0.84). The estimated haplotype means follow this pattern and are correlated with the estimated effects for the *timeless cis*-eQTL in most cases (average absolute value correlation: 0.65; min: 0.03; max: 0.99). The gene *timeless (tim)* is expressed in the adult central nervous system [37] and is involved in transcriptional regulation of circadian rhythm [38].

**Table 2:** *trans*-eQTL regulating multiple genes.

	# eQTL	chr	lower (Mb)	upper (Mb)	# genes in interval	# genes in interval with *cis*-eQTL
TQTLA	16	*2L*	3.22	3.65	53	14
TQTLB	11	*2R*	8.33	9.12	119	50

Not all genes in the TQTLA interval are included in our expression set. For example, some genes may have been dropped due to the presence of SNPs in probes, or were not included in the Nimblegen probe set to begin with. For TQTLA, 23 genes in the interval are not represented in the expression set. However, none of these genes are associated with any terms involving circadian rhythm, regulation of gene expression, or transcription (http://FlyBase.org) [39], and we therefore do not consider any of these likely candidate genes.

The genes associated with TQTLB are enriched for several GO terms including compound eye pigmentation (2/11 genes; P = 0.005), the umbrella term: single-organism metabolic process (6/11 genes; P = 0.007), and several specific metabolic process terms: tryptophan metabolic process (2/11 genes; P = 0.008), indolalkylamine metabolic process (2/11 genes; P = 0.0008), indole-containing compound metabolic process (2/11 genes; P = 0.002), aromatic amino acid family metabolic process (2/11 genes; P = 0.006). Once again we performed PCA to create a composite variable. *sugarbabe* (*sug*) was the gene most highly correlated with the TQTLB composite variable (r = −0.63) and does have a significant *cis*-eQTL. All other genes in the interval had a correlation with an absolute value of less than 0.4. Loadings were again fairly even (absolute value range for all other genes: 0.08–0.39). Pairwise correlations between the transcripts associated with TQTLB and the expression measures in the interval showed *sugarbabe* to be most highly correlated in all cases except two: gene *CG5321* and gene *CG6834* (absolute value of correlation range for all other genes: 0.40–0.52). These two genes were also the two with the lowest loading values on the composite variable. The correlation between the estimated haplotype effects for the *cis*-eQTL for *sugarbabe*, and the effects for the *trans*-eQTLs were moderate (mean absolute value correlation: 0.24; min: 0.005; max: 0.44). The gene *sugarbabe (sug)* is expressed in the adult head [37], is involved in regulation of transcription [40], is involved in regulation of response to starvation [41], and is part of the insulin-like growth factor signaling pathway [41]. The 21 genes not included in the interval are not associated with any terms involving metabolism, regulation of gene expression, or transcription (http://FlyBase.org) [39].

We have identified two *trans* hotspots, and, in both cases, we were able to use our expression dataset to narrow the causative gene to a single likely candidate gene. Previous eQTL studies have identified many more *trans* hotspots that regulate many more genes (hundreds or thousands) than our two identified hotspots (TQTLA: 16 genes; TQTLB: 11 genes; e.g. [27], [42], reviewed in [24], [26]). However, while some of these global regulators of gene expression have been confirmed as true signals, most notably in yeast [43], [44], Kang et al. [43] show how hotspots can result from confounding factors such as batch effects. In our dataset, we employed PCA to correct for possible batch effects [45]. This method has been shown to increase power to detect eQTL [29], [45], [46], however, it makes identifying even true *trans* global regulators impossible. The signal that results from a global regulator is statistically indistinguishable from an unmeasured batch effect. In addition, even true global regulators can confound the detection of other true eQTLs, and correcting for these true global regulators increases the power to detect these other associations [43], [45]. It is possible to distinguish true *trans* hotspots from batch effects using biological replicates [43], but for our study we chose to maximize the number of RILs rather than increase replication to maximize our statistical power to map eQTL. As a result, we are unable to detect many *trans* hotspots in this study. However, our stringent statistical correction does give us increased confidence that the eQTL we do identify are indeed true signals.

### Most eQTLs are multiallelic

**Figure 5:**
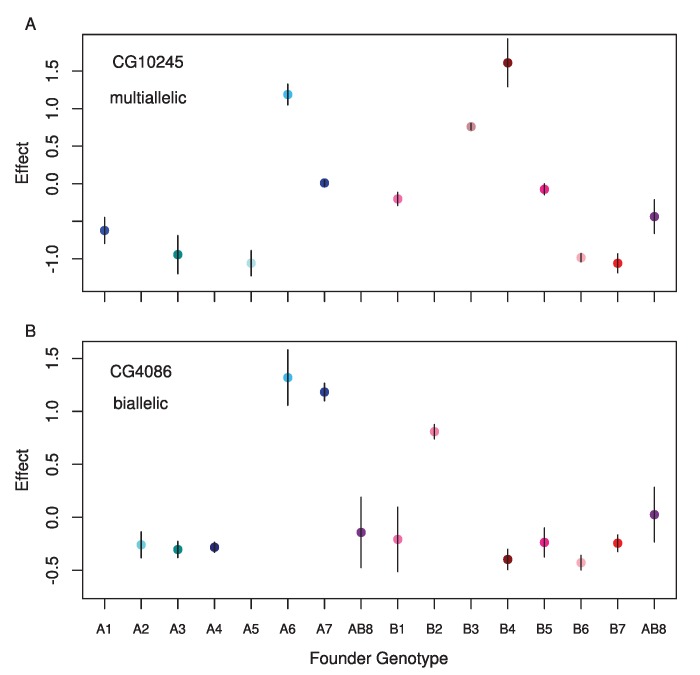
Standardized estimated means for each founder genotype for single observed *cis*-eQTL where A) the full model is the best model, and B) a two allele model is the best model. The gene name for each is displayed in the upper left corner.

**Figure 6:**
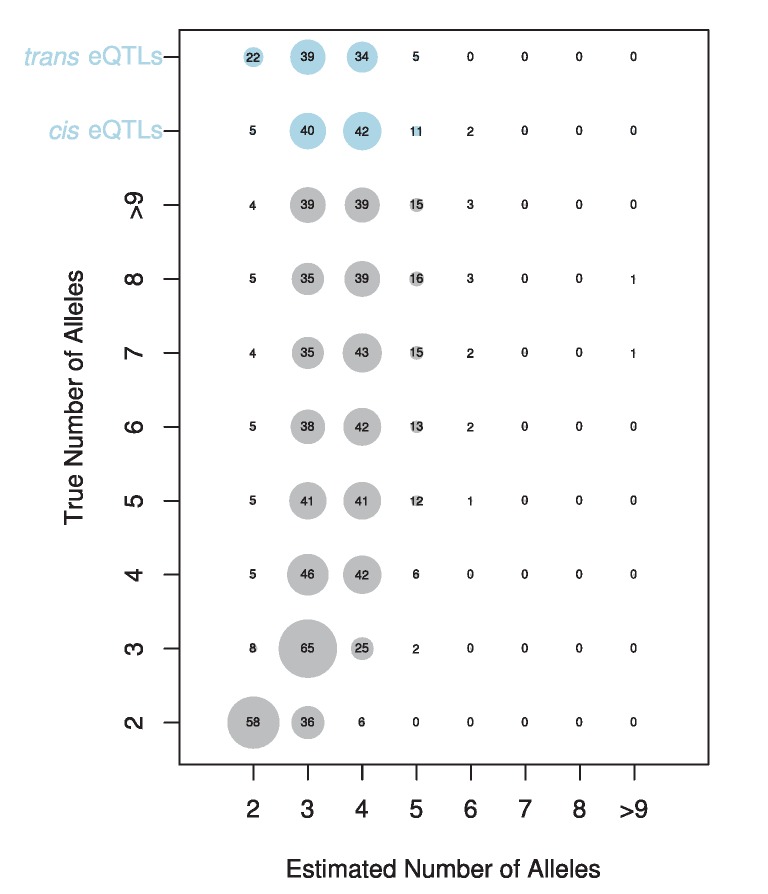
Estimated number of alleles for simulated (grey circles) and observed data (blue circles). The true number of alleles versus the estimated number of alleles is displayed for simulated data. The size of each circle and the number displayed denotes the percentage of times each number of alleles is estimated for a given true number of alleles. The estimated number of alleles for our *cis*- and *trans*-eQTL are shown at the top of the plot.

The vast majority of our eQTLs appear to be multiallelic (Figure S1). In 95% of cases, the number of alleles estimated at *cis*-eQTL was 3 or greater. For *trans*-eQTL this percentage was somewhat lower, at 78%. Figure 5 shows an example of an eQTL where the best model is a two allele model and of an eQTL where the full haplotype model is the best model. In cases where we estimated multiple alleles, we were able to explain additional phenotypic variance compared to the best two allele model (Figure S2), sometimes as much as an additional 27%. We investigated our ability to accurately estimate the number of alleles by performing a simulation designed to provide the highest power to distinguish between different alleles (see methods). Our simulation revealed that our estimator underestimates the number of alleles in 63% of cases, correctly estimates the true number of alleles in 26% of cases, and overestimates the number of alleles in 10% of cases (Figure 6). This bias toward underestimating the number of alleles gets increasingly severe as the true number of alleles increases. Our simulations with a lower effect size (5%) and normally distributed allelic effects both resulted in an even stronger bias toward underestimating the true number of alleles. Our allele number distribution for *cis*-eQTLs is no doubt composed of a mixture of eQTLs of varied numbers of true alleles. Overall, it is closest to the distribution we obtain for a simulation of ∼5 alleles. So while most of our estimates for *cis*-eQTL are for 3–4 alleles, many may be determined by many more alleles.

Our results indicate widespread allelic heterogeneity for both *cis*- and *trans*-eQTLs. The focus of mapping studies is often to identify the single causative variant underlying a significant signal, the implicit assumption being that the causative loci are biallelic. *cis*-eQTL in particular, with their large effects, are thought to be more likely than other traits to have a simple genetic architecture and be biallelic [22], [28]–[30]. Baud et al. [22] found some support for this idea when comparing a two allele model to the full haplotype model in hippocampus eQTLs in the heterogeneous stock mouse resource [32]. They found that in 97% of cases, the two allele model was superior for *cis*-eQTLs while *trans*-eQTLs were more likely to be multiallelic [22]. However, in contrast to these findings, *cis*-eQTLs have been found to be multiallelic in *Drosophila* [47], *Arabidopsis* [42], and humans [31], [48]. Our results strongly confirm the result of multiallelism in *Drosophila* with 95% of *cis*-eQTLs estimated to be due to 3 or more alleles. This result indicates that in *Drosophila*, widespread allelic heterogeneity exists at one of the most basic levels of genetic variation: *cis*-regulatory variation.

Widespread allelic heterogeneity is one potential explanation for the missing heritability problem in the study of complex traits. Allelic heterogeneity presents a statistical challenge for GWAS [7]. GWAS utilize natural populations and interrogate each SNP (or other specific variant) for association with the phenotype of interest. At the single gene level, it is difficult to distinguish between simple linkage disequilibrium between a single causative variant and other, nearby neutral SNPs, and multiple independent causative SNPs. If GWAS focus only on the strongest association at a locus, in the presence of allelic heterogeneity that individual variant will account for less of the variation than the entire gene, causing the effect of the locus to be underestimated [7]. In this respect, haplotype-based mapping approaches, such as the one described here, have an advantage because entire haplotypes (and thus an entire set of causative variants associated with a single gene) are tested together. The effect size associated with the causative gene will tend to be larger and easier to detect in this framework. This effect, combined with the more favorable frequencies of alleles in linkage based panels could explain why these studies tend to explain very large proportions of the heritable variation [9], [21], [49], while GWAS grapple with large amounts of missing heritability. However, one drawback of current haplotype-based methods is that they do not have single gene resolution and therefore identifying the causative gene within the QTL interval can be a significant challenge. Furthermore, while identifying the causative loci under allelic heterogeneity is easier with haplotype based methods, the subsequent identification of the causative SNPs within the loci is made much more complicated by heterogeneity [17], [18], [50].

Allelic heterogeneity is typical for Mendelian diseases (http://www.omim.org/) and it has been suggested as the likely model for quantitative traits [51]. There is a growing body of empirical [2], [17], [22], [31], [42], [47], [50] and theoretical [7] support for this idea. For example, one of the largest GWA studies found support for allelic heterogeneity for human height by identifying several cases of multiple SNPs likely associated with the same gene [2]. Even age related macular degeneration, the first successful GWA study [52], has subsequently been shown to harbor multiple functional alleles [53]–[56]. Our results should therefore not be surprising. However, they do suggest the community should focus on developing experimental designs and analytical methods, e.g., [7], that function well under a model of allelic heterogeneity.

## Methods

### Mapping population

We used RILs from the DSPR (http://FlyRILs.org) to map genome-wide expression variation. The DSPR has been described in detail previously. Complete details of the development of the DSPR, founder whole genome re-sequencing, and RIL genotyping are described in [17]. The development of the hidden Markov model to infer the mosaic structure of the RILs and the power and mapping resolution of the DSPR for QTL mapping are described in [18]. Briefly, the DSPR is a multi-founder advanced intercross panel consisting of a set of over 1700 RILs of *Drosophila melanogaster*. Two 8-way synthetic populations (pA and pB) were created from two independent sets of 7 inbred founder lines (A1–A7 or B1–B7) with one additional line (AB8) shared by both populations. Each synthetic population was maintained as two independent replicate subpopulations (pA1 and pA2 or pB1 and pB2), kept at a large population size, and allowed to freely recombine for 50 generations. At generation 50, each subpopulation gave rise to ∼500 RILs via 25 generations of full-sib mating. The genomes of the original fifteen inbred founder lines have been completely re-sequenced, and the complete underlying founder haplotype structure of all RILs in the panel has been determined via Restriction-Associated DNA (RAD) sequencing along with a hidden Markov model (HMM).

In order to avoid potentially mapping QTL for inbreeding depression, we phenotyped *trans*-heterozygote F1 individuals from crosses between pA females and pB males. The crosses were done to maintain the subpopulation structure by crossing pA1 to pB2 and pA2 to pB1. In both cases, we arbitrarily crossed pA and pB RILs with the same line number (*i.e.*, pA1_1_*pB2_1_, …, pA1_n_*pB2_n_, pA2_1_*pB1_1_, …, pA2_n_*pB1_n_). For each of 596 crosses, we generated 4–6 replicate cross vials containing 10 virgin pA females and 10 pB males and cleared the adults after 24–48 hours to maintain roughly equal larval density across experimental vials. Both the inbred RIL parents and the experimental *trans*-heterozygous cross progeny were raised on standard cornmeal-yeast-molasses media at 25°C, 50% relative humidity, and on a 12∶12 light∶dark regime.

### RNA isolation and arrays

Progeny from each cross vial were allowed to emerge and mate in the source vial for 2–4 days. Then 250–300 females were harvested over CO_2_ from the multiple replicate vials. Since we did not isolate virgin females on eclosion, females are very likely mated. These experimental females were kept for 24 hours in fresh vials to minimize any effects of the anesthesia before the heads were isolated (3–5 days old). Heads were removed by transferring the females without anesthesia to a 50 ml conical bottom centrifuge tube, freezing in liquid nitrogen, vigorously vortexing, and sieving using dry ice-chilled brass analytical sieves (mesh sizes 0.0165 and 0.0278 inches), separating heads from bodies and from legs and wings. Head samples were stored at −80°C until RNA isolation.

We did not have any technical or biological replicates aside from the effect of pooling 250–300 individuals, collected from multiple source vials, for each sample. This was intentional because we are mainly interested in the variance among RILs. There were two exceptions to this lack of replication. Crosses A1.299×B2.299 and A1.350×B2.350 were prepared independently twice.

RNA was isolated using TRIzol Reagent (Life Technologies), cleaned up using RNeasy Mini spin columns (Qiagen), concentrated—if necessary—using a vacuum centrifuge, and shipped to the Carver Center for Genomics Microarray Center at the University of Iowa for cDNA synthesis and array hybridization. We used Nimblegen 12×135 K arrays to assay genome-wide gene expression. These arrays assay 16,637 transcripts with eight 60 bp probes per transcript. Each array holds 12 different crosses.

### Data analysis pipeline

All data analysis was performed in R [57]. Initially, we performed standard quantile normalization and corrected for background effects using the normalize and backgroundCorrect functions in the oligo package to correct for any overall array effects [58]–[61]. We then created a custom probe-to-transcript map using the most recent version of the CDS file available at FlyBase (v. 5.48). We blasted all probe sequences against the CDS, requiring an exact match [62], [63]. We eliminated any probe sequences without an exact 60 bp match to a transcript (6842 probes). We did not require a unique match given many transcripts from the same gene share portions of their sequences. Thus a single probe can correspond to multiple transcripts.

Single nucleotide polymorphisms in probe sequences are known to affect array hybridization and thus expression measurement [64]–[68]. We took advantage of the availability of full genome sequences for all 15 founder lines to identify SNPs within probe sequences. We first updated the alignment and SNP calling for the founder re-sequencing data using the Burrows-Wheeler Aligner (BWA) [69] with the following switches: -m 50000000 -R 5000, followed by the SAMtools [70] mpileup command (the initial alignment used Mosaik and a custom SNP caller, see [17]) to obtain an accurate, comprehensive list of SNPs in the founder lines (http://FlyRILs.org/Data, Release 3). We also applied the following filters: 1) at least one founder was fixed for the minor allele and at least three founders were fixed for the major allele (given a coverage of 10×), 2) minimum overall coverage of 90 (5 per sample), and 3) maximum overall coverage of 3600. A large proportion of our probe sequences contained SNPs segregating in the set of DSPR founder lines. Because we have the full genome sequences *in silico* of all RILs in the panel, we were able to identify all positions in probes that are SNPs in our RIL panel and test for the effect of each SNP on the expression measurement. We discarded any probes containing multiple SNPs (22018 probes). For probes containing a single SNP, we used the haplotype probabilities from the hidden Markov model to infer the probability each RIL harbored the minor allele and assigned a genotype value to each cross by adding the paternal and maternal probabilities. In the case of perfect certainty, genotype values are: 2 = AA, 1 = Aa, and 0 = aa. We then tested for the effect of the SNP on the expression measurement by fitting the following model:
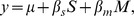
where *y* is the expression measurement, *S* is subpopulation, *M* is the cross genotype at the marker, and *β_s_* and *β_m_* are the corresponding effect estimates. We then eliminated all probes with a p-value less than 0.05 (21141 probes).

Following re-mapping of probes and elimination of probes with SNPs affecting expression, transcripts were associated with a variable number of probes instead of each transcript being associated with exactly 8 probes as in the original NimbleGen array design. We eliminated any transcript associated with fewer than four probes. Next, we performed standard RMA using the basicRMA function in the oligo package [61] to combine probe-specific data and generate a single expression measure per transcript. Many genes are associated with multiple transcripts. Whether the expression of different transcripts can be independently assessed is dependent on how many probes uniquely map to each transcript. We calculated pairwise correlations between each transcript in each set of transcripts associated with a single gene. If all of the pairwise correlations between the set of transcripts were > = 0.95, we used the average expression for the gene. Otherwise, we mapped each transcript separately. We will refer to all expression measures (including those averaged across transcripts for a single gene) simply as transcripts for clarity.

We followed the methods of [29], [46] and used principal components analysis (PCA) to minimize batch effects [45] and increase our power to detect QTL. Following quantile normalization of each transcript to coerce each transcript distribution to be normal, we performed PCA on the entire set of transcripts. We selected the first 10 principal components to correct our expression measurements. The percentage of the variance explained by each remaining principal component was below 1% (Figure S3). We then fit the following model
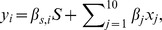
where *y_i_* is the *i*th expression measurement, *S* is subpopulation, *x_j_* is the *j*th principal component, and *β_s,i_* and *β_j_* are the corresponding effect estimates. We used the resulting residuals for the remaining analyses. We performed an additional round of quantile normalization on these residuals to ensure normality.

We estimated the narrow-sense heritabilities for all transcripts by fitting a linear mixed model using the polygenic function in the GenABEL package [71]. Briefly, the model includes a random effect polygenic term whose variance is determined by the kinship matrix between RIL crosses. We calculated the kinship matrix using the genome-wide haplotype assignments resulting from the HMM. At each position spaced every 0.025 cM, we calculated the probability of identity by decent and averaged these across the genome to obtain the relationship coefficient. Our kinship matrix is thus estimated over genetic distance. We then used the polygenic function to calculate heritabilities for each transcript [71].

To map eQTLs, we first selected transcripts expressed above background levels. We utilized the two replicated samples, A1.299×B2.299 and A1.350×B2.350, to identify the point where measurements were less repeatable and excluded all transcripts with expression levels below this point (Figure S4). This cutoff excluded approximately 23% of transcripts. For all included transcripts, we performed haplotype-based genome scans by fitting the following model at regularly spaced positions every 10 KB across the genome (11768 positions; http://FlyRILs.org/Data, Release 3).
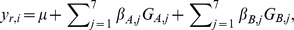
where *y_r,i_* is the ith transcript, *μ* is the grand mean, *G_A,j_* are the genotype probabilities for the jth paternal RIL, *G_B,j_* are the genotype probabilities for the jth maternal RIL, and *β_A,j_*, and *β_B,j_* are the corresponding effect estimates. Because we assayed only females, the model for the *X* chromosome is the same as for the autosomes. At each position, we calculated the *F*-statistic for the overall effect of genotype and obtained LOD scores.

To identify the statistical significance threshold, we performed 1000 permutations of the expression measures [72]. The entire set of expression measures was permuted together to maintain the correlation structure in the dataset. We used these permutations to determine a conservative genome-wide, experiment-wise 5% significance threshold (threshold = 14.99). We also determined a separate threshold for *cis*-eQTL. We defined *cis*-eQTL as QTL occurring within 1.5 cM of the transcription start [18] site for each transcript (1.5 cM is our typical confidence interval width). To define a *cis*-only threshold, we only included the LOD scores for the positions within 1.5 cM of the transcription start for each gene (threshold = 14.4).

We identified all peaks with LOD scores exceeding the above-defined thresholds. When multiple nearby peaks were identified, we determined whether their 3 LOD drop intervals overlapped, and, if so, only the peak with the highest LOD score was retained. We expect 3 LOD drops to be a conservative estimate of the 95% confidence interval. Standard 2 LOD drops have been shown to be overly narrow for pA×pB cross designs [18]. It should be noted however, that confidence intervals on QTL locations are not true 95% confidence intervals and effect size, sample size, and the number of haplotypes in the model affect the degree of coverage. We also calculated Bayes credible intervals, for which 95% coverage tends to be more consistent [73], [74].

In a pA×pB cross, a mapped QTL may be due to genomic variation at that position in only one population or in both. We identified peaks associated with only a single population using Akaike's Information Criterion (AIC). We calculated the AIC for three models: pA alone, pB alone, and pA & pB. The smallest AIC indicates the model with the best fit. Thus any cases in which the lowest AIC resulted from a reduced model, the QTL peak was concluded to be due to variation in a single population.

We identified *trans*-eQTLs influencing multiple transcripts by estimating the *trans*-eQTL density across the genome using a 500 kb sliding window with a step size of 1 kb. Our estimate of density included only unique genes, not transcripts to avoid counting multiple transcripts associated with a single gene as independent events. If *trans*-eQTL density in a window exceeded the density expected by chance under a Poisson distribution, we concluded it was a significant *trans* hotspot. This threshold for a Poisson distribution given the total number of *trans*-eQTLs (147), the window size (500 kb), the size of the genome tested (118 Mb) and the Bonferonni corrected P-value threshold (117,741 tests; P = 4.2×10^−7^) is a *trans*-eQTL density greater than 6. We delineated the size of these hotspot regions as the lowermost and uppermost confidence interval bound for any *trans*-eQTL peak included in a window exceeding a density of 6.

Our initial scan identified 3 *trans* hotspots but upon further investigation, we determined one to be a false signal resulting from a single gene family. All of the eQTL peaks associated with this hotspot represent 13 members of a single gene family located on the *X* chromosome: *Stellate* (*Ste*). In addition, members of this family also occur at an unlocalized region in the heterochromatin on the *X* chromosome. The “*trans-*” eQTL we map regulating this family is located at the very tip of the *X* chromosome, making it very likely we are tagging this heterochromatic location of *Stellate* members, and it is in fact an additional *cis* effect. In fact, all thirteen members show two peaks, one *cis* peak and a second “*trans*” peak at the tip of the *X*, indicating most of our probes for these genes are tagging multiple members of this gene family. In addition, *Stellate* is expressed in adult males and involved in spermatogenesis (http://FlyBase.org) [39]. It is likely we are seeing high expression due to large numbers of copies of gene family members (∼200 copies) [75]. We therefore excluded this *trans* hotspot.

### Estimating the number of alleles at eQTLs

We estimated the number of alleles at each eQTL using a model comparison technique similar to the method employed by Yalcin et al. [76] and Baud et al. [22] The major difference in our approach is that we consider models with more than 2 alleles and do not restrict our analysis to specific SNPs in the QTL interval. The merge analysis employed by Baud et al. [22] considered all two allele models associated with a single SNP within the QTL interval. We simply assign different alleles to different haplotypes without those necessarily corresponding to SNPs in the interval. This method also allows us to consider models with several alleles. For each eQTL, at the peak position, we fit all possible models for different numbers of alleles, fitting a maximum of 11337 models at each eQTL. We first estimated the haplotype means at the peak, sorted these means, and then fit all possible models that did not change the order of the haplotype means for 2, 3, 4, 5, 6, 7, 8, and 16 (the full model allowing different estimates for AB8 in pA RILs and AB8 in pB RILs) alleles (Figure S5). We only included haplotypes at the peak that occurred at least 5 times (at a probability of greater than 95%) in our set of crosses. Haplotypes at lower frequencies lead to inaccurate estimates of haplotype means with large standard errors. For each possible allele grouping, individual founder haplotype probabilities in each allele group were summed to obtain a probability each RIL harbored each allele group. For example, if haplotypes A3 and A5 are grouped as a single allele named allele 1, and the probabilities a given RIL cross harbors the A3 or A5 haplotype are 0.90 and 0.03 respectively, then the probability that RIL cross harbors allele 1 is 0.93 (i.e., the probability the RIL cross harbors either A3 OR A5 and thus allele 1). Alleles were only combined within pA and within pB given that the pA and pB sets of probabilities are independent. The model fit was as follows:
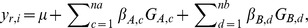
where *y_r,i_* is the ith transcript, *μ* is the grand mean, *na* is the number of pA allele groupings, *nb* is the number of pB allele groupings, *G_A,c_* are the genotype probabilities for the *c*th paternal allele group, *G_B,d_* are the genotype probabilities for the *d*th maternal allele group, and *β_A,c_*, and *β_B,d_* are the corresponding effect estimates. The model with the lowest P-value was chosen as the best model and the number of alleles associated with this model was recorded. We also explored using Akaike's information criterion (AIC) to choose the best model, however simulations revealed a higher error rate using AIC (see below). Table S3 provides hard coded genotype assignments for all RIL crosses at all significant eQTL.

### Simulation

To test our method of estimating the number of alleles associated with QTL, we simulated QTL stemming from between 2 and 15 different alleles and subsequently estimated the number of alleles using the model comparison methodology described above. We intentionally set up this simulation to make distinguishing different alleles as easy as possible. We performed 1000 iterations for each of 2, 3, 4, 5, 6, 7, 8 and 15 alleles (the full model assuming the same effect for AB8 in the pA and pB panels). For each iteration, we randomly selected 600 pA RILs and 600 pB RILs from the DSPR panel and randomly paired them to create pA-pB crosses. We then simulated a QTL in this set of RIL crosses at a randomly selected position in the genome with the chosen number of alleles. We assigned the different alleles equal effects, because we found equal effects gave higher power to distinguish different alleles compared to pulling effects from a normal distribution (Figure S6). For example, for a four allele model each founder haplotype was randomly assigned an effect of 1, 2, 3, or, 4. We assumed an additive model to calculate a genetic effect for each cross. We generated a set of random normal deviates N(μ = 0, 
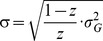
) to correspond to environmental variance where *z* = the percent of the phenotypic variance explained by the QTL and 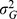 is the genetic variance at the QTL. The percent of the total phenotypic variance explained by the QTL was randomly chosen from our observed distribution of phenotypic variance explained by *cis*-eQTLs. These effects tend to be quite large, however, we found large effects lead to higher power to distinguish different alleles (Figure S7). We then estimated the number of alleles at our simulated QTL as described above. We used two methods to determine the best model: 1) the model with the lowest P-value, and 2) the model with the lowest AIC. Our results showed the method using P-values had a greater accuracy (P-value method: 26% accuracy; AIC method: 19% accuracy). More importantly, the AIC method overestimates the true number of alleles more often, estimating more than two alleles in 83% of cases when the true number of alleles is two (Table S2). We prefer the method that is more conservative, meaning it has a greater tendency to underestimate rather than overestimate the number of alleles, and we therefore use the P-value method in all subsequent analysis (Figure S8). Complete sensitivity information for the different methods and the different simulation models can be seen in Figures S5, S6, S7 and in Table S2.

## Supporting Information

Figure S1 A) Histogram of the estimated number of alleles using the lowest P-value to determine the best model (see Methods). B) Histogram of the estimated number of alleles using AIC to determine the best model.
(EPS)

Figure S2 Boxplot of the additional percent variance explained by the best multiallelic model compared to the best two allele model for cases where a multiallelic model is best. The x-axis shows the number of alleles estimated in the best multiallelic model. The black center line of the box is the median additional percent variance explained for each estimated number of alleles (lower edge of the box is the first quartile, upper edge is the third quartile, whiskers extend to 1.5 times the interquartile range).
(PDF)

Figure S3 The proportion of variance accounted for by the first 50 eigentraits (principal components) following a principal components analysis on all transcript expression measures. The vertical dotted line denotes the cut off at the 10th principal component. Only these first 10 principal components were statistically corrected for in the subsequent analyses.
(PDF)

Figure S4 The correlation between replicate measures of transcript expression for RIL cross A: A1.299×B2.299 and C: A1.350×B2.350. The absolute difference between the replicates versus the average expression for each transcript is shown for RIL cross B: A1.299×B2.299 and D: A1.350×B2.350.
(PDF)

Figure S5 Diagram of the procedure to estimate the number of alleles at a QTL. Estimated haplotype means are sorted and then all possible models are tested. The various models are shown for the 3 allele case. The model with the lowest p value is chosen as the best model and the associated number of alleles is our estimate of the number of alleles at the QTL.
(PDF)

Figure S6 The true number of alleles versus the estimated number of alleles for a simulation where the genetic effect for each allele is sampled from a normal distribution. The size of each circle and the number displayed denotes the percentage of times each number of alleles is estimated for a given true number of alleles. The estimated number of alleles for our *cis*- and *trans*-eQTL are shown at the top of the plot in blue.
(PDF)

Figure S7 The true number of alleles versus the estimated number of alleles for a simulation with a constant effect size of 5% for the simulated QTL. The size of each circle and the number displayed denotes the percentage of times each number of alleles is estimated for a given true number of alleles. The estimated number of alleles for our *cis*- and *trans*-eQTL are shown at the top of the plot in blue.
(PDF)

Figure S8 The true number of alleles versus the estimated number of alleles for a simulation identical to that described in the main text but with AIC determining the best model instead of the lowest P-value. The size of each circle and the number displayed denotes the percentage of times each number of alleles is estimated for a given true number of alleles. The estimated number of alleles for our *cis*- and *trans*-eQTL using the AIC method are shown at the top of the plot in blue.
(PDF)

Table S1 Complete details for all eQTL. Columns are as follows: Name = eQTL identifier, TID = transcript identifier (CG name) for transcripts mapped separately, gene identifier otherwise, GID = gene identifier (CG name), chr = chromosome location of eQTL peak, peakp = physical position of eQTL peak, peaklpL = lower confidence interval bound using 3 LOD drop (physical position), peakupL = upper confidence interval bound using 3 LOD drop (physical position), peaklpB = lower confidence interval bound using Bayesian credible interval (physical position), peakupB = upper confidence interval bound using Bayesian credible interval (physical position), peakg = genetic position of eQTL peak, peaklgL = lower confidence interval bound using 3 LOD drop (genetic position), peakugL = upper confidence interval bound using 3 LOD drop (genetic position), peaklgB = lower confidence interval bound using Bayesian credible interval (genetic position), peakugB = upper confidence interval bound using Bayesian credible interval (genetic position), LOD = LOD score at eQTL peak, Pvar = percent phenotypic variance explained by eQTL peak, h2 = heritability of transcript abundance, psdist = physical distance to transcript start site, gsdist = genetic distance to transcript start site, cis = true/false for whether eQTL is cis, GlocC = chromosomal location of transcript, GlocP = physical location of transcript start site, GlocG = genetic location of transcript start site.
(TXT)

Table S2 Sensitivity of the minimum P-value and AIC method of estimating different alleles for different simulation models. For each, the probability of estimating 2 or more alleles given a true value of 2 or more alleles is displayed.
(DOC)

Table S3 Hard coded founder genotype assignments at all significant eQTL. Each RIL at each eQTL peak is assigned the most likely founder genotype, given the probability is greater than 0.95. This corresponds to a 2 digit number with the assignment from the population A RIL and the population B RIL. E.g. the number 24 indicates that RIL cross has an A2B4 genotype. If the highest founder genotype probability is less than 0.95 it is coded as uncertain. The number 9 indicates an uncertain assignment. Column names for columns 5 to 601 are the maternal RIL ID. The paternal RIL is the RIL with the matching number in the corresponding subpopulation (see Methods). Other columns are: Name = eQTL identifier, TID = transcript identifier (CG name) for transcripts mapped separately, gene identifier otherwise, GID = gene identifier (CG name), chr = chromosome location of eQTL peak.
(ZIP)

## References

[1] Zuk O, Hechter E, Sunyaev SR, Lander ES (2012) The mystery of missing heritability: Genetic interactions create phantom heritability. Proceedings of the National Academy of Sciences 109: 1193–1198 10.1073/pnas.1119675109 PMC3268279 22223662

[2] Lango Allen H, Estrada K, Lettre G, Berndt SI, Weedon MN, et al (2010) Hundreds of variants clustered in genomic loci and biological pathways affect human height. Nature 467: 832–838 10.1038/nature09410 20881960 PMC2955183

[3] Yang J, Benyamin B, Mcevoy BP, Gordon S, Henders AK, et al (2010) Common SNPs explain a large proportion of the heritability for human height. Nat Genet 42: 565–569 10.1038/ng.608 20562875 PMC3232052

[4] Rockman MV (2012) The QTN program and the alleles that matter for evolution: all that's gold does not glitter. Evolution 66: 1–17 10.1111/j.1558-5646.2011.01486.x 22220860 PMC3386609

[5] Pritchard J (2001) Are rare variants responsible for susceptibility to complex diseases? Am J Hum Genet 69: 124–137. 11404818 10.1086/321272 PMC1226027

[6] Bansal V, Libiger O, Torkamani A, Schork NJ (2010) Statistical analysis strategies for association studies involving rare variants. Nat Rev Genet 11: 773–785 10.1038/nrg2867 20940738 PMC3743540

[7] Thornton KR, Foran AJ, Long AD (2013) Properties and Modeling of GWAS when Complex Disease Risk Is Due to Non-Complementing, Deleterious Mutations in Genes of Large Effect. PLoS Genet 9: e1003258 10.1371/journal.pgen.1003258.s011 23437004 PMC3578756

[8] Churchill GA, Airey DC, Allayee H, Angel JM, Attie AD, et al (2004) The Collaborative Cross, a community resource for the genetic analysis of complex traits. Nat Genet 36: 1133–1137 10.1038/ng1104-1133 15514660

[9] Aylor DL, Valdar W, Foulds-Mathes W, Buus RJ, Verdugo RA, et al (2011) Genetic analysis of complex traits in the emerging Collaborative Cross. Genome Res 21: 1213–1222 10.1101/gr.111310.110 21406540 PMC3149489

[10] Philip VM, Sokoloff G, Ackert-Bicknell CL, Striz M, Branstetter L, et al (2011) Genetic analysis in the Collaborative Cross breeding population. Genome Res 21: 1223–1238 10.1101/gr.113886.110 21734011 PMC3149490

[11] Kover PX, Valdar W, Trakalo J, Scarcelli N, Ehrenreich IM, et al (2009) A Multiparent Advanced Generation Inter-Cross to Fine-Map Quantitative Traits in *Arabidopsis thaliana* . PLoS Genet 5: e1000551 10.1371/journal.pgen.1000551 19593375 PMC2700969

[12] Huang X, Paulo M-J, Boer M, Effgen S, Keizer P, et al (2011) Analysis of natural allelic variation in *Arabidopsis* using a multiparent recombinant inbred line population. P Natl Acad Sci Usa 108: 4488–4493 10.1073/pnas.1100465108 PMC3060268 21368205

[13] Yu J, Holland JB, McMullen MD, Buckler ES (2008) Genetic design and statistical power of nested association mapping in maize. Genetics 178: 539–551 10.1534/genetics.107.074245 18202393 PMC2206100

[14] Buckler ES, Holland JB, Bradbury PJ, Acharya CB, Brown PJ, et al (2009) The Genetic Architecture of Maize Flowering Time. Science 325: 714–718 10.1126/science.1174276 19661422

[15] McMullen MD, Kresovich S, Sanchez Villeda H, Bradbury P, Li H, et al (2009) Genetic Properties of the Maize Nested Association Mapping Population. Science 325: 737–740 10.1126/science.1174320 19661427

[16] Li H, Bradbury P, Ersoz E, Buckler ES, Wang J (2011) Joint QTL Linkage Mapping for Multiple-Cross Mating Design Sharing One Common Parent. PLOS ONE 6: e17573 10.1371/journal.pone.0017573 21423655 PMC3057965

[17] King EG, Merkes CM, McNeil CL, Hoofer SR, Sen S, et al (2012) Genetic dissection of a model complex trait using the *Drosophila* Synthetic Population Resource. Genome Res 22: 1558–1566 10.1101/gr.134031.111 22496517 PMC3409269

[18] King EG, Macdonald SJ, Long AD (2012) Properties and power of the *Drosophila* Synthetic Population Resource for the routine dissection of complex traits. Genetics 191: 935–949 10.1534/genetics.112.138537 22505626 PMC3389985

[19] Mackay TFC, Richards S, Gibbs RA (2008) Proposal to Sequence a *Drosophila* Genetic Reference Panel: A Community Resource for the Study of Genotypic and Phenotypic Variation: 1–32. Available at https://www.genome.gov/Pages/Research/Sequencing/SeqProposals/DrosophilaSeq.pdf

[20] Mackay TFC, Richards S, Stone EA, Barbadilla A, Ayroles JF, et al (2012) The *Drosophila melanogaster* Genetic Reference Panel. Nature 482: 173–178 10.1038/nature10811 22318601 PMC3683990

[21] Bloom JS, Ehrenreich IM, Loo WT, Lite T-LV, Kruglyak L (2013) Finding the sources of missing heritability in a yeast cross. Nature 494: 234–237 10.1038/nature11867 23376951 PMC4001867

[22] Baud A, Hermsen R, Guryev V, Stridh P, Graham D, et al (2013) Combined sequence-based and genetic mapping analysis of complex traits in outbred rats. Nat Genet 45: 767–775 10.1038/ng.2644 23708188 PMC3821058

[23] Cookson W, Liang L, Abecasis GR, Moffatt M, Lathrop M (2009) Mapping complex disease traits with global gene expression. Nat Rev Genet 10: 184–194 10.1038/nrg2537 19223927 PMC4550035

[24] Gibson G, Weir B (2005) The quantitative genetics of transcription. Trends Genet 21: 616–623 10.1016/j.tig.2005.08.010 16154229

[25] Sun G, Schliekelman P (2011) A Genetical Genomics Approach to Genome Scans Increases Power for QTL Mapping. Genetics 187: 939–953 10.1534/genetics.110.123968 21196521 PMC3063683

[26] Gilad Y, Rifkin SA, Pritchard JK (2008) Revealing the architecture of gene regulation: the promise of eQTL studies. Trends Genet 24: 408–415 10.1016/j.tig.2008.06.001 18597885 PMC2583071

[27] Ehrenreich IM, Gerke JP, Kruglyak L (2010) Genetic Dissection of Complex Traits in Yeast: Insights from Studies of Gene Expression and Other Phenotypes in the BYxRM Cross. Cold Spring Harbor Symposia on Quantitative Biology 74: 145–153 10.1101/sqb.2009.74.013 PMC2888688 19734204

[28] Veyrieras J-B, Kudaravalli S, Kim SY, Dermitzakis ET, Gilad Y, et al (2008) High-Resolution Mapping of Expression-QTLs Yields Insight into Human Gene Regulation. PLoS Genet 4: e1000214 10.1371/journal.pgen.1000214.s023 18846210 PMC2556086

[29] Gaffney DJ, Veyrieras J-B, Degner JF, Pique-Regi R, Pai AA, et al (2012) Dissecting the regulatory architecture of gene expression QTLs. Genome Biol 13: R7 10.1186/gb-2012-13-1-r7 22293038 PMC3334587

[30] Degner JF, Pai AA, Pique-Regi R, Veyrieras J-B, Gaffney DJ, et al (2012) DNase I sensitivity QTLs are a major determinant of human expression variation. Nature 482: 390–394 10.1038/nature10808 22307276 PMC3501342

[31] Lappalainen T, Sammeth M, Friedländer MR, Hoen PACT, Monlong J, et al (2013) Transcriptome and genome sequencing uncovers functional variation in humans. Nature 501: 506–511 10.1038/nature12531 24037378 PMC3918453

[32] Huang G-J, Shifman S, Valdar W, Johannesson M, Yalcin B, et al (2009) High resolution mapping of expression QTLs in heterogeneous stock mice in multiple tissues. Genome Res 19: 1133–1140 10.1101/gr.088120.108 19376938 PMC2694476

[33] Yang J, Ferreira T, Morris AP, Medland SE, Madden PAF, et al (2012) Conditional and joint multiple-SNP analysis of GWAS summary statistics identifies addtional variants influencing comlex traits. Nat Genet 44: 369–375 10.1038/ng.2213 22426310 PMC3593158

[34] Xu S (2003) Theoretical basis of the Beavis effect. Genetics 165: 2259–2268. 14704201 10.1093/genetics/165.4.2259 PMC1462909

[35] Edgar R, Domrachev M, Lash AE (2002) Gene Expression Omnibus: NCBI gene expression and hybridization array data repository. Nucleic Acids Research 30: 207–210. 11752295 10.1093/nar/30.1.207 PMC99122

[36] Boyle EI, Weng S, Gollub J, Jin H, BOTSTEIN D, et al (2004) GO::TermFinder–open source software for accessing Gene Ontology information and finding significantly enriched Gene Ontology terms associated with a list of genes. Bioinformatics 20: 3710–3715 10.1093/bioinformatics/bth456 15297299 PMC3037731

[37] Robinson SW, Herzyk P, Dow JAT, Leader DP (2012) FlyAtlas: database of gene expression in the tissues of Drosophila melanogaster. Nucleic Acids Research 41: D744–D750 10.1093/nar/gks1141 23203866 PMC3531048

[38] Koh K, Evans JM, Hendricks JC, Sehgal A (2006) A *Drosophila* model for age-associated changes in sleep:wake cycles. P Natl Acad Sci Usa 103: 13843–13847 10.1073/pnas.0605903103 PMC1564207 16938867

[39] Marygold SJ, Leyland PC, Seal RL, Goodman JL, Thurmond J, et al (2012) FlyBase: improvements to the bibliography. Nucleic Acids Research 41: D751–D757 10.1093/nar/gks1024 23125371 PMC3531214

[40] Zinke I, Schütz CS, Katzenberger JD, Bauer M, Pankratz MJ (2002) Nutrient control of gene expression in Drosophila: microarray analysis of starvation and sugar-dependent response. EMBO J 21: 6162–6173. 12426388 10.1093/emboj/cdf600 PMC137192

[41] Varghese J, Lim SF, Cohen SM (2010) *Drosophila* miR-14 regulates insulin production and metabolism through its target, sugarbabe. Genes & Development 24: 2748–2753 10.1101/gad.1995910 21159815 PMC3003191

[42] Zhang X, Cal AJ, Borevitz JO (2011) Genetic architecture of regulatory variation in Arabidopsis thaliana. Genome Res 21: 725–733 10.1101/gr.115337.110 21467266 PMC3083089

[43] Kang HM, Ye C, Eskin E (2008) Accurate Discovery of Expression Quantitative Trait Loci Under Confounding From Spurious and Genuine Regulatory Hotspots. Genetics 180: 1909–1925 10.1534/genetics.108.094201 18791227 PMC2600931

[44] Smith EN, Kruglyak L (2008) Gene–Environment Interaction in Yeast Gene Expression. PLoS Biol 6: e83 10.1371/journal.pbio.0060083.st004 18416601 PMC2292755

[45] Leek JT, Storey JD (2007) Capturing heterogeneity in gene expression studies by surrogate variable analysis. PLoS Genet 3: 1724–1735 10.1371/journal.pgen.0030161 17907809 PMC1994707

[46] Pickrell JK, Marioni JC, Pai AA, Degner JF, Engelhardt BE, et al (2010) Understanding mechanisms underlying human gene expression variation with RNA sequencing. Nature 464: 768–772 10.1038/nature08872 20220758 PMC3089435

[47] Gruber JD, Long AD (2008) Cis-regulatory Variation Is Typically Polyallelic in *Drosophila* . Genetics 181: 661–670 Available: http://www.genetics.org/cgi/doi/10.1534/genetics.108.098459. 19064705 10.1534/genetics.108.098459 PMC2644954

[48] Powell JE, Henders AK, McRae AF, Kim J, Hemani G, et al (2013) Congruence of Additive and Non-Additive Effects on Gene Expression Estimated from Pedigree and SNP Data. PLoS Genet 9: e1003502 10.1371/journal.pgen.1003502.s016 23696747 PMC3656157

[49] Nuzhdin SV, Pasyukova E, Dilda C, Zeng Z, Mackay TFC (1997) Sex-specific quantitative trait loci affecting longevity in Drosophila melanogaster. P Natl Acad Sci Usa 94: 9734–9739. 10.1073/pnas.94.18.9734 PMC23259 9275193

[50] Kislukhin G, King EG, Walters KN, Macdonald SJ, Long AD (2013) The Genetic Architecture of Methotrexate Toxicity Is Similar in *Drosophila melanogaster* and Humans. G3 (Bethesda) 3: 1301–1310 10.1534/g3.113.006619 23733889 PMC3737169

[51] McClellan J, King M-C (2010) Genetic Heterogeneity in Human Disease. Cell 141: 210–217 10.1016/j.cell.2010.03.032 20403315

[52] Klein RJ (2005) Complement Factor H Polymorphism in Age-Related Macular Degeneration. Science 308: 385–389 10.1126/science.1109557 15761122 PMC1512523

[53] Hughes AE, Orr N, Esfandiary H, Diaz-Torres M, Goodship T, et al (2006) A common CFH haplotype, with deletion of CFHR1 and CFHR3, is associated with lower risk of age-related macular degeneration. Nat Genet 38: 1173–1177 10.1038/ng1890 16998489

[54] Li M, Atmaca-Sonmez P, Othman M, Branham KEH, Khanna R, et al (2006) CFH haplotypes without the Y402H coding variant show strong association with susceptibility to age-related macular degeneration. Nat Genet 38: 1049–1054 10.1038/ng1871 16936733 PMC1941700

[55] Maller J, George S, Purcell S, Fagerness J, Altshuler D, et al (2006) Common variation in three genes, including a noncoding variant in CFH, strongly influences risk of age-related macular degeneration. Nat Genet 38: 1055–1059 10.1038/ng1873 16936732

[56] Swaroop A, Branham KE, Chen W, Abecasis G (2007) Genetic susceptibility to age-related macular degeneration: a paradigm for dissecting complex disease traits. Human Molecular Genetics 16: R174–R182 10.1093/hmg/ddm212 17911160

[57] R Core Team (2013) R: a language and environment for statistical computing. Available: http://www.R-project.org/.

[58] Bolstad BM, Irizarry RA, Astrand M, Speed TP (2003) A comparison of normalization methods for high density oligonucleotide array data based on variance and bias. Bioinformatics 19: 185–193. 12538238 10.1093/bioinformatics/19.2.185

[59] Irizarry RA, Bolstad BM, Collin F, Cope LM, Hobbs B, et al (2003) Summaries of Affymetrix GeneChip probe level data. Nucleic Acids Research 31: e15. 12582260 10.1093/nar/gng015 PMC150247

[60] Irizarry RA, Hobbs B, Collin F, Beazer-Barclay YD, Antonellis KJ, et al (2003) Exploration, normalization, and summaries of high density oligonucleotide array probe level data. Biostatistics 4: 249–264 10.1093/biostatistics/4.2.249 12925520

[61] Carvalho BS, Irizarry RA (2010) A framework for oligonucleotide microarray preprocessing. Bioinformatics 26: 2363–2367 10.1093/bioinformatics/btq431 20688976 PMC2944196

[62] Altschul SF, Gish W, Miller W, Myers EW, Lipman DJ (1990) Basic local alignment search tool. J Mol Biol 215: 403–410 10.1016/S0022-2836(05)80360-2 2231712

[63] Camacho C, Coulouris G, Avagyan V, Ma N, Papadopoulos J, et al (2009) BLAST+: architecture and applications. BMC Bioinformatics 10: 421 10.1186/1471-2105-10-421 20003500 PMC2803857

[64] Alberts R, Terpstra P, Li Y, Breitling R, Nap J-P, et al (2007) Sequence polymorphisms cause many false cis eQTLs. PLOS ONE 2: e622 10.1371/journal.pone.0000622 17637838 PMC1906859

[65] Benovoy D, Kwan T, Majewski J (2008) Effect of polymorphisms within probe-target sequences on olignonucleotide microarray experiments. Nucleic Acids Research 36: 4417–4423 10.1093/nar/gkn409 18596082 PMC2490733

[66] Chen L, Page GP, Mehta T, Feng R, Cui X (2009) Single nucleotide polymorphisms affect both cis- and trans-eQTLs. Genomics 93: 501–508 10.1016/j.ygeno.2009.01.011 19248827 PMC4041081

[67] Ciobanu DC, Lu L, Mozhui K, Wang X, Jagalur M, et al (2010) Detection, validation, and downstream analysis of allelic variation in gene expression. Genetics 184: 119–128 10.1534/genetics.109.107474 19884314 PMC2802080

[68] Ramasamy A, Trabzuni D, Gibbs JR, Dillman A, Hernandez DG, et al (2013) Resolving the polymorphism-in-probe problem is critical for correct interpretation of expression QTL studies. Nucleic Acids Research 41: e88 10.1093/nar/gkt069 23435227 PMC3627570

[69] Li H, Durbin R (2009) Fast and accurate short read alignment with Burrows-Wheeler transform. Bioinformatics 25: 1754–1760 10.1093/bioinformatics/btp324 19451168 PMC2705234

[70] Li H, Handsaker B, Wysoker A, Fennell T, Ruan J, et al (2009) The Sequence Alignment/Map format and SAMtools. Bioinformatics 25: 2078–2079 10.1093/bioinformatics/btp352 19505943 PMC2723002

[71] Aulchenko YS, de Koning D-J, Haley C (2007) Genomewide rapid association using mixed model and regression: A fast and simple method for genomewide pedigree-based quantitative trait loci association analysis. Genetics 177: 577–585 10.1534/genetics.107.075614 17660554 PMC2013682

[72] Churchill GA, Doerge RW (1994) Empirical threshold values for quantitative trait mapping. Genetics 138: 963–971. 7851788 10.1093/genetics/138.3.963 PMC1206241

[73] Manichaikul A, Dupuis J, Sen S, Broman KW (2006) Poor performance of bootstrap confidence intervals for the location of a quantitative trait locus. Genetics 174: 481–489 Available: http://www.genetics.org/cgi/doi/10.1534/genetics.106.061549. 16783000 10.1534/genetics.106.061549 PMC1569776

[74] Broman KW, Sen S (2009) A Guide to QTL Mapping with R/qtl. Springer New York.

[75] Lyckegaard EM, Clark AG (1989) Ribosomal DNA and Stellate gene copy number variation on the Y chromosome of *Drosophila melanogaster* . P Natl Acad Sci Usa 86: 1944–1948. 10.1073/pnas.86.6.1944 PMC286821 2494656

[76] Yalcin B (2005) Using Progenitor Strain Information to Identify Quantitative Trait Nucleotides in Outbred Mice. Genetics 171: 673–681 10.1534/genetics.104.028902 16085706 PMC1456780

