# Supplementary figures and images for: Genetic Dissection of the Drosophila melanogaster Female Head Transcriptome Reveals Widespread Allelic Heterogeneity
Source: PLoS Genet. 2014 May 8;10(5):e1004322. doi: 10.1371/journal.pgen.1004322 (PMC4014434; doi:10.1371/journal.pgen.1004322)

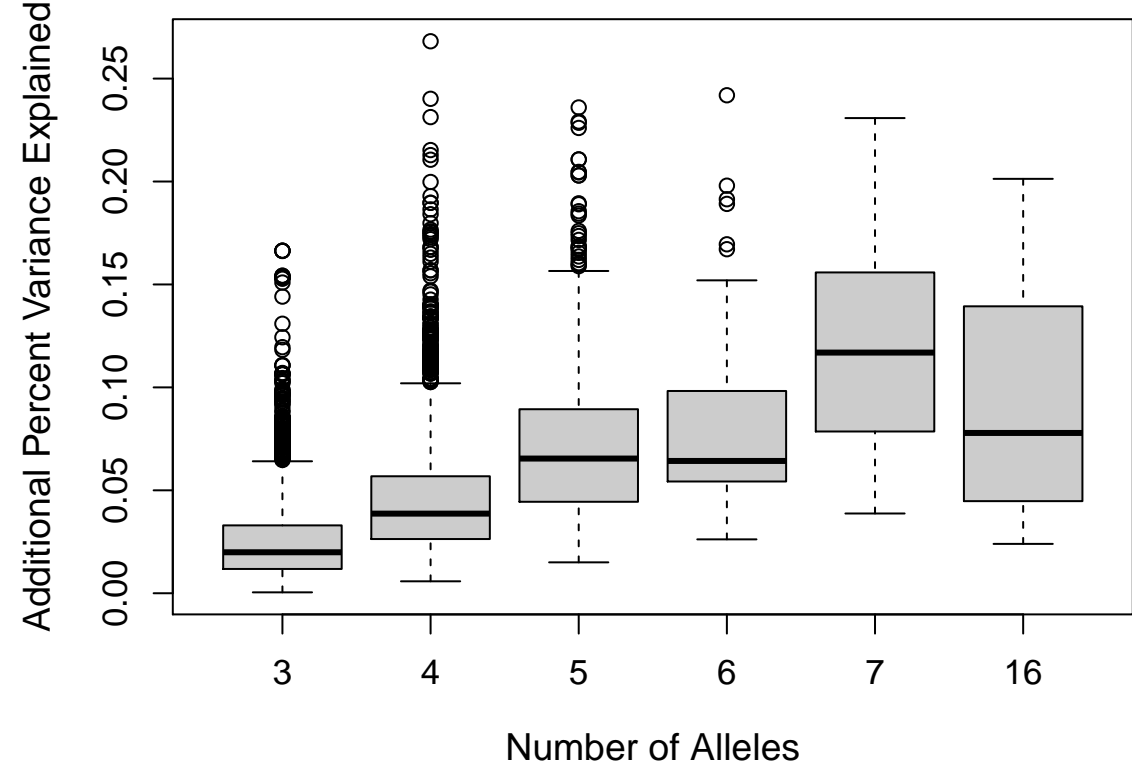

Supplement: Figure S2 — Boxplot of the additional percent variance explained by the best multiallelic model compared to the best two allele model for cases where a multiallelic model is best. The x-axis shows the number of alleles estimated in the best multiallelic model. The black center line of the box is the median additional percent variance explained for each estimated number of alleles (lower edge of the box is the first quartile, upper edge is the third quartile, whiskers extend to 1.5 times the interquartile range). (PDF) [file pgen.1004322.s002.pdf]

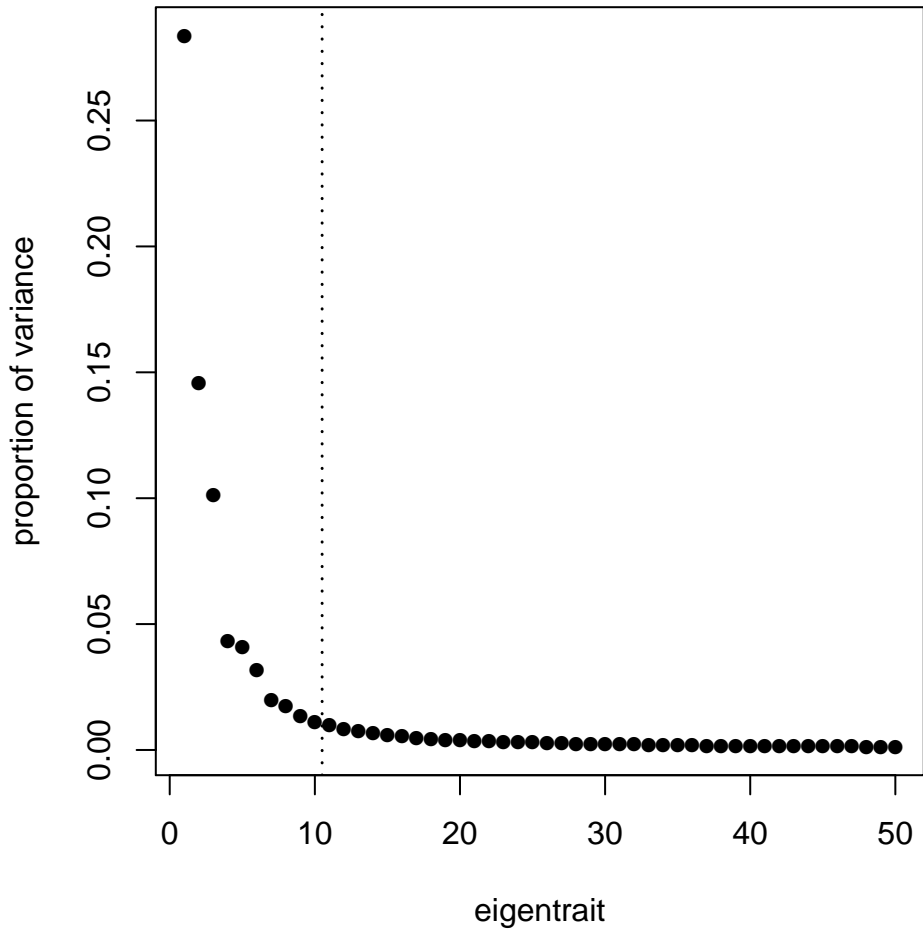

Supplement: Figure S3 — The proportion of variance accounted for by the first 50 eigentraits (principal components) following a principal components analysis on all transcript expression measures. The vertical dotted line denotes the cut off at the 10th principal component. Only these first 10 principal components were statistically corrected for in the subsequent analyses. (PDF) [file pgen.1004322.s003.pdf]

**A**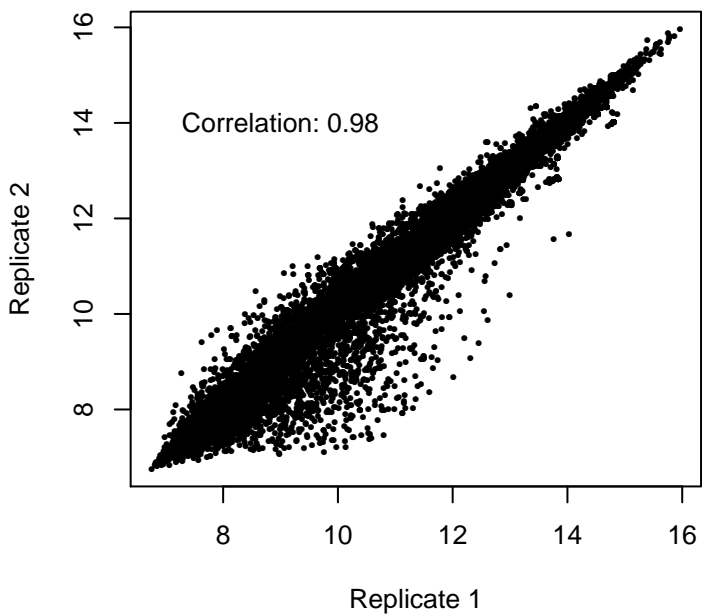**B**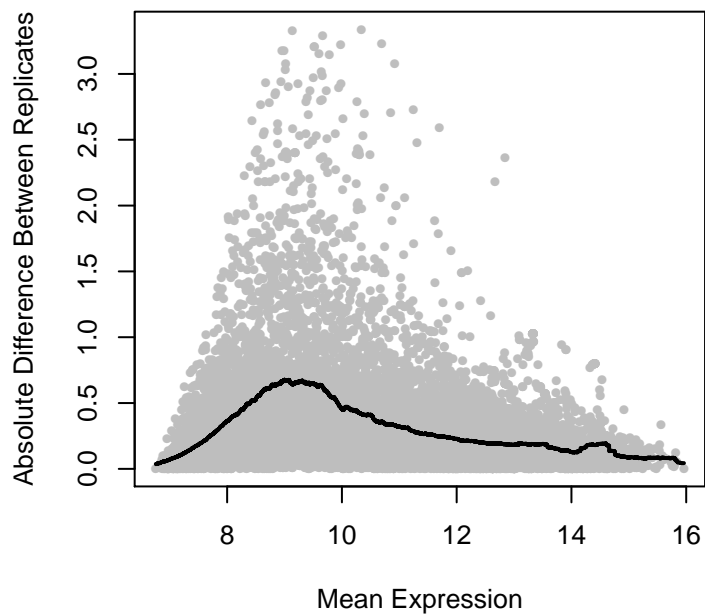**C**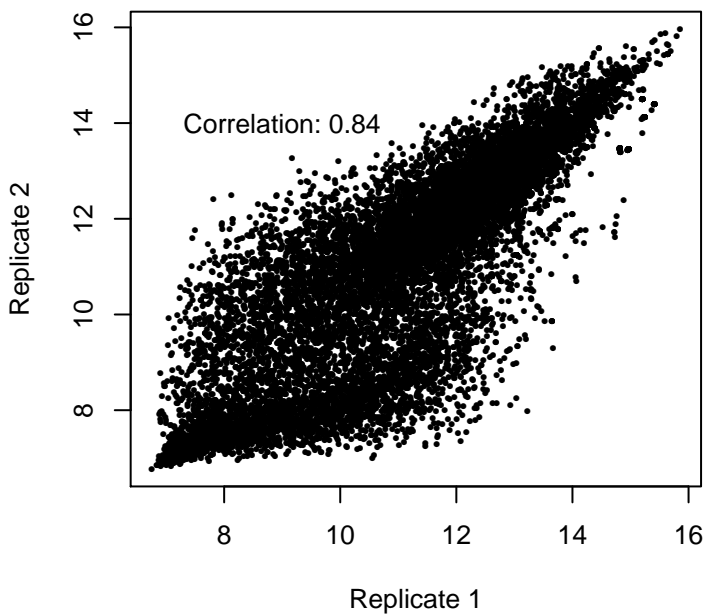**D**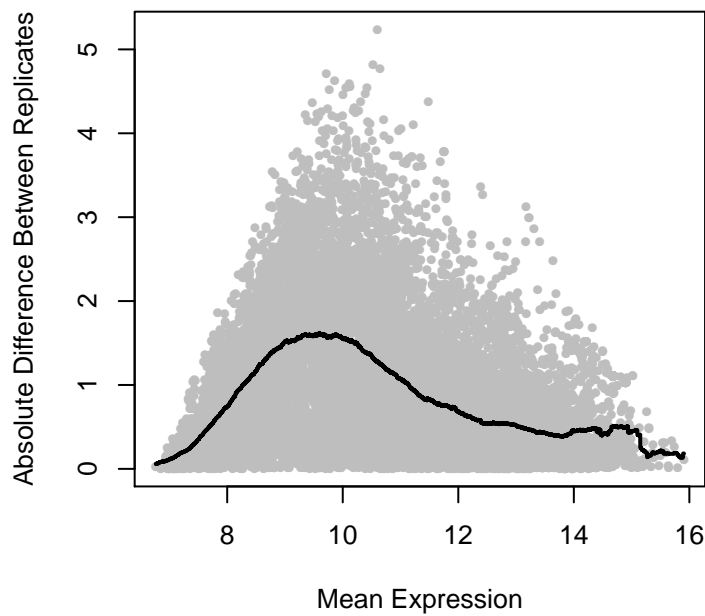

Supplement: Figure S4 — The correlation between replicate measures of transcript expression for RIL cross A: A1.299×B2.299 and C: A1.350×B2.350. The absolute difference between the replicates versus the average expression for each transcript is shown for RIL cross B: A1.299×B2.299 and D: A1.350×B2.350. (PDF) [file pgen.1004322.s004.pdf]

*trans* eQTLs

*cis* eQTLs

True Number of Alleles

>9  
8  
7  
6  
5  
4  
3  
2

2 3 4 5 6 7 8 >9

Estimated Number of Alleles

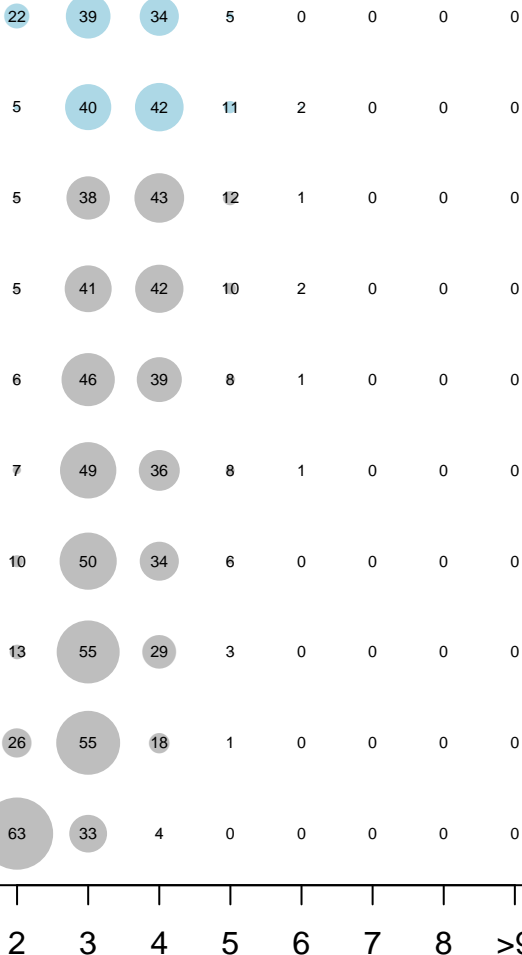

Supplement: Figure S6 — The true number of alleles versus the estimated number of alleles for a simulation where the genetic effect for each allele is sampled from a normal distribution. The size of each circle and the number displayed denotes the percentage of times each number of alleles is estimated for a given true number of alleles. The estimated number of alleles for our cis- and trans-eQTL are shown at the top of the plot in blue. (PDF) [file pgen.1004322.s006.pdf]

*trans* eQTLs

*cis* eQTLs

True Number of Alleles

2 3 4 5 6 7 8 >9

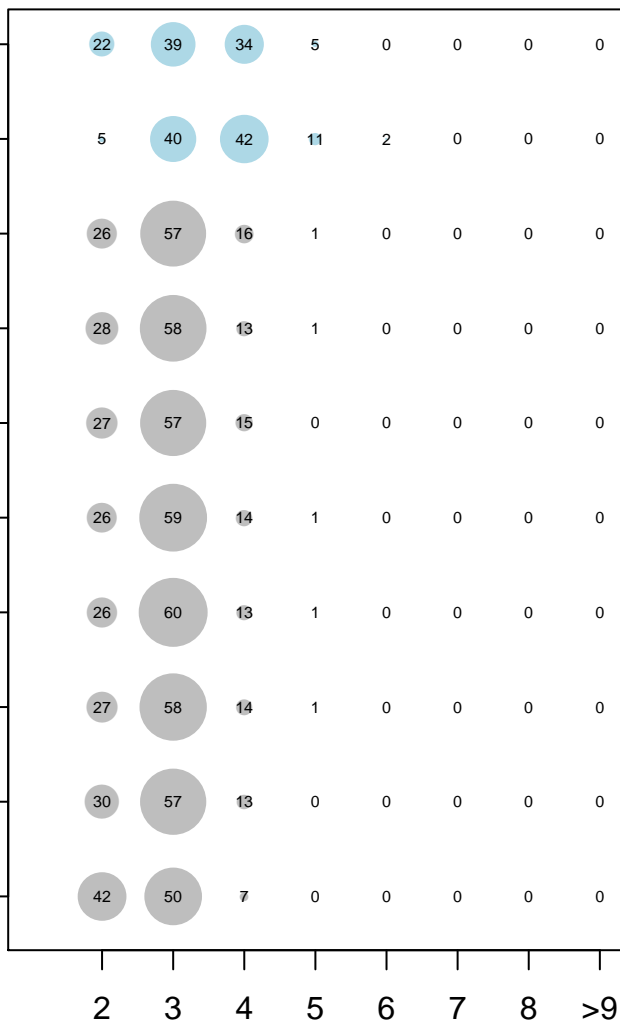

Estimated Number of Alleles

Supplement: Figure S7 — The true number of alleles versus the estimated number of alleles for a simulation with a constant effect size of 5% for the simulated QTL. The size of each circle and the number displayed denotes the percentage of times each number of alleles is estimated for a given true number of alleles. The estimated number of alleles for our cis- and trans-eQTL are shown at the top of the plot in blue. (PDF) [file pgen.1004322.s007.pdf]

*trans* eQTLs

*cis* eQTLs

True Number of Alleles

>9  
8  
7  
6  
5  
4  
3  
2

2 3 4 5 6 7 8 >9

Estimated Number of Alleles

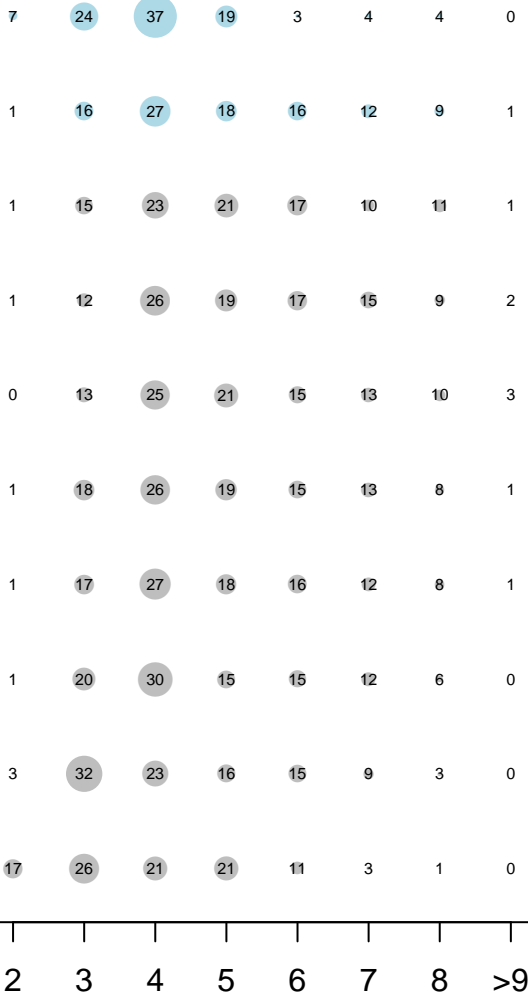

Supplement: Figure S8 — The true number of alleles versus the estimated number of alleles for a simulation identical to that described in the main text but with AIC determining the best model instead of the lowest P-value. The size of each circle and the number displayed denotes the percentage of times each number of alleles is estimated for a given true number of alleles. The estimated number of alleles for our cis- and trans-eQTL using the AIC method are shown at the top of the plot in blue. (PDF) [file pgen.1004322.s008.pdf]
