# Supplementary material for: Genetic Dissection of the Drosophila melanogaster Female Head Transcriptome Reveals Widespread Allelic Heterogeneity
Source: PLoS Genet. 2014 May 8;10(5):e1004322. doi: 10.1371/journal.pgen.1004322 (PMC4014434; doi:10.1371/journal.pgen.1004322)
Supplement: Figure S5 — Diagram of the procedure to estimate the number of alleles at a QTL. Estimated haplotype means are sorted and then all possible models are tested. The various models are shown for the 3 allele case. The model with the lowest p value is chosen as the best model and the associated number of alleles is our estimate of the number of alleles at the QTL. (PDF) [file pgen.1004322.s005.pdf]

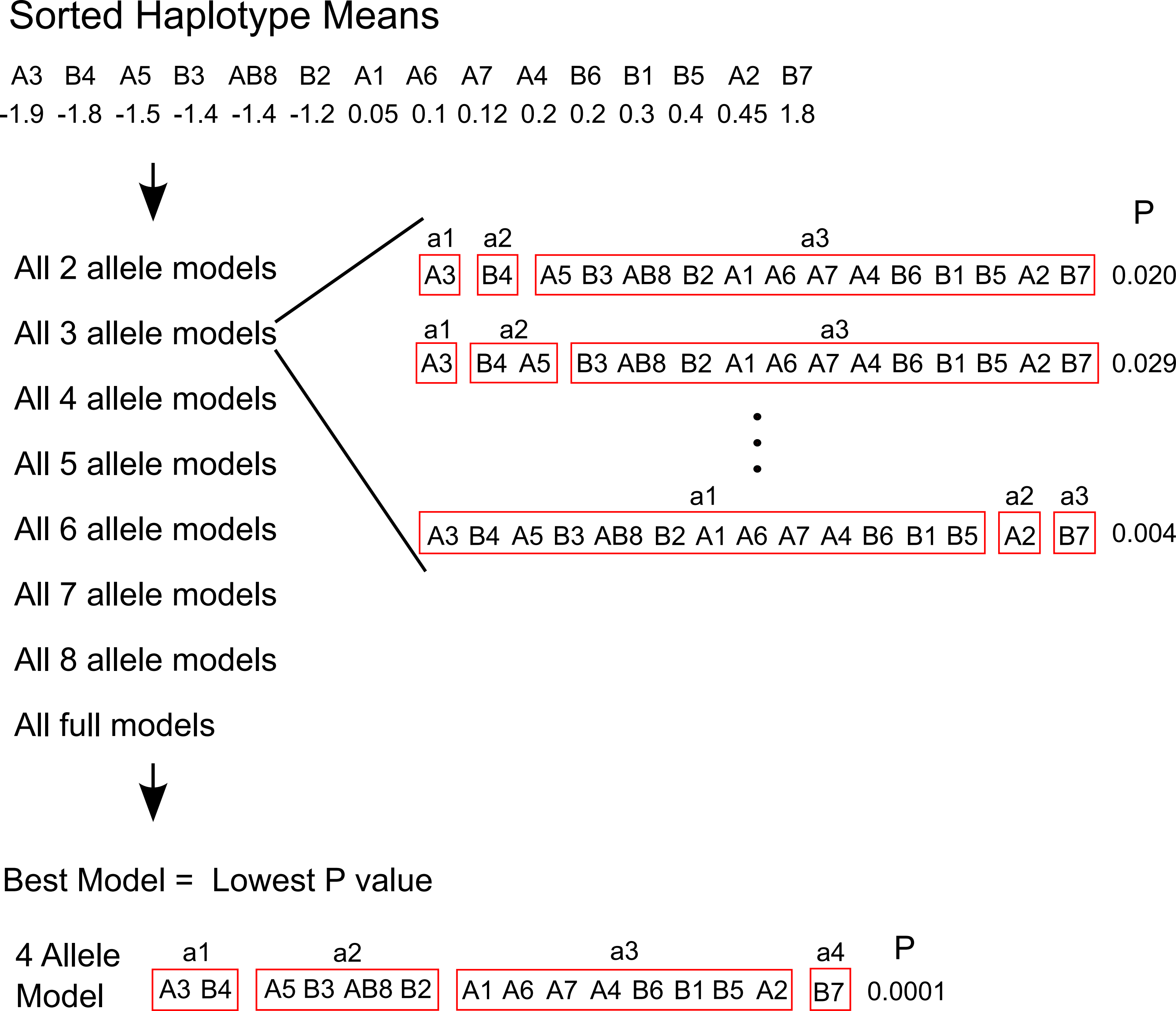
Sorted Haplotype Means
A3 B4 A5 B3 AB8 B2 A1 A6 A7 A4 B6 B1 B5 A2 B7
-1.9 -1.8 -1.5 -1.4 -1.4 -1.2 0.05 0.1 0.12 0.2 0.2 0.3 0.4 0.45 1.8
All 2 allele models
All 3 allele models
All 4 allele models
All 5 allele models
All 6 allele models
All 7 allele models
All 8 allele models
All full models
P
a1 a2 a3
A3 B4 A5 B3 AB8 B2 A1 A6 A7 A4 B6 B1 B5 A2 B7 0.020
a1 a2 a3
A3 B4 A5 B3 AB8 B2 A1 A6 A7 A4 B6 B1 B5 A2 B7 0.029
a1 a2 a3
A3 B4 A5 B3 AB8 B2 A1 A6 A7 A4 B6 B1 B5 A2 B7 0.004
Best Model = Lowest P value
4 Allele Model
a1 a2 a3 a4 P
A3 B4 A5 B3 AB8 B2 A1 A6 A7 A4 B6 B1 B5 A2 B7 0.0001
