# Supplementary material for: Genetic Dissection of the Drosophila melanogaster Female Head Transcriptome Reveals Widespread Allelic Heterogeneity
Source: PLoS Genet. 2014 May 8;10(5):e1004322. doi: 10.1371/journal.pgen.1004322 (PMC4014434; doi:10.1371/journal.pgen.1004322)
Supplement: Table S2 — Sensitivity of the minimum P-value and AIC method of estimating different alleles for different simulation models. For each, the probability of estimating 2 or more alleles given a true value of 2 or more alleles is displayed. (DOC) [file pgen.1004322.s010.doc]

|  | | Estimated Number | True Number | |
| --- | --- | --- | --- | --- |
| Standard Model | |  | Two | More |
|  | min(P-value) |  |
|  |  | Two | 0.58 | 0.05 |
|  |  | More | 0.42 | 0.95 |
|  | AIC |  |  |  |
|  |  | Two | 0.16 | 0.01 |
|  |  | More | 0.83 | 0.99 |
| Unequal Allelic Effects | |  |  |  |
|  | min(P-value) |  |  |  |
|  |  | Two | 0.63 | 0.12 |
|  |  | More | 0.37 | 0.88 |
|  | AIC |  |  |  |
|  |  | Two | 0.19 | 0.03 |
|  |  | More | 0.81 | 0.97 |
| 5% Effect Size | |  |  |  |
|  | min(P-value) |  |  |  |
|  |  | Two | 0.42 | 0.27 |
|  |  | More | 0.58 | 0.73 |
|  | AIC |  |  |  |
|  |  | Two | 0.29 | 0.17 |
|  |  | More | 0.71 | 0.83 |
